# Twenty-five years of briefings in bioinformatics

**DOI:** 10.1093/bib/bbag237

**Published:** 2026-05-19

**Authors:** Martin Bishop

**Affiliations:** University of Cambridge, Cambridge, United Kingdom

It is a privilege to reflect on 25 years of *Briefings in Bioinformatics* (BIB). We launched at the turn of the millennium, at a time when the bioinformatics landscape was a rapidly evolving frontier. Our first issues in 2000 appeared as the Human Genome Project approached its initial completion in 2003. In those days, ‘bioinformatics’ was often understood in relatively narrow terms: sequence analysis, database construction, and the development of algorithms for alignment, annotation, and gene prediction. Yet even then, it was clear that the field stood on deeper historical foundations: structural studies by physical techniques predated sequence determination, and protein sequencing itself preceded nucleic acid sequencing. Bioinformatics did not emerge in isolation; it was the convergence of multiple traditions in biology, chemistry, mathematics, and computation. I grew up with a deep sense of wonder at the natural world; it is a particular privilege to have witnessed how, over a lifetime, that wonder has been enriched by an increasingly detailed understanding of the molecular processes that underlie it.

Serving as Editor-in-Chief for 23 years, from the journal’s birth until my retirement in 2023, allowed me a front-row seat to a scientific transformation. Initially a quarterly published by Henry Stewart Publications, the journal was taken over by Oxford University Press in 2006 and soon became bimonthly, with a steadily increasing scope and page count. From the outset, our aim was not only to document the progress of the field but to provide genuine ‘briefings’: reviews written in a way that did not assume deep computational expertise, enabling experimental biologists to incorporate mathematical and statistical approaches into their work. In this sense, BIB has always served as a bridge between disciplines.

In the early years, the central object of study was the genome, and the central challenge was scale—how to store, search, and interpret rapidly accumulating sequence data. Review articles reflected this landscape: they synthesized emerging tools, compared methods, and helped a growing community navigate what was then a fast-moving but still relatively bounded field. Foundational tools such as BLAST and FASTA for sequence similarity searching, and CLUSTAL and later MAFFT for multiple sequence alignment, became indispensable. The transition from Sanger sequencing to next-generation sequencing (NGS) marked a decisive turning point. What had been a specialized activity became an essential pillar of biological research, as the volume and diversity of data expanded by orders of magnitude.

The subsequent shift from the genome to the transcriptome and proteome broadened the field’s scope. High-throughput technologies enabled the systematic study of gene expression, protein abundance, and post-translational modification. At the same time, new computational challenges emerged: normalization, statistical modelling, and the integration of heterogeneous datasets. The rise of epigenomics added another layer, highlighting that sequence alone is not destiny, but is modulated by chromatin state and regulatory context.

For many years, predicting protein structure from sequence remained an aspirational goal. Progress through homology modelling, threading, and fragment-based approaches was steady but incremental. The recent advent of deep learning–based methods has altered that landscape profoundly. While it would be premature to declare the problem fully solved, it is now possible to obtain highly accurate structural models for a vast number of proteins. This has shifted the centre of gravity once again: from sequence to structure, and increasingly to function in context. Structure-informed approaches are reshaping areas such as drug discovery and protein engineering, moving the discussion from ‘What is the structure?’ to ‘What can we do with it therapeutically?’

Perhaps the most significant conceptual shift during my tenure has been the move towards systems biology. It became increasingly clear that a catalogue of components is insufficient; understanding arises from interactions. Bioinformatics has therefore expanded to encompass network analysis, pathway modelling, and integrative approaches that attempt to reconcile data across multiple levels—genomic, transcriptomic, proteomic, and beyond. The ambition, still very much in progress, is a mechanistic understanding of cellular processes at the molecular level.

In recent years, several developments have further reshaped the field. Single-cell technologies have moved us beyond bulk averages to the heterogeneity of individual cells, revealing new layers of biological complexity. Metagenomics and microbiome research have extended bioinformatics from the study of individual organisms to entire communities. Advances in cloud computing and high-performance computing have changed how data are stored, shared, and analysed, making large-scale studies more accessible while also raising new questions about standardization and reproducibility.

At the same time, the emergence of machine learning and, more recently, large language models is influencing how we analyse and interpret biological data. These approaches hold great promise, particularly in pattern recognition and hypothesis generation, but they also require careful validation and critical assessment. As in earlier phases of the field, the challenge is not only innovation but also robust evaluation, interpretability, and responsible use.

When I stepped down in 2023, handing over to Shuangge Ma, I did so with a sense of continuity as well as pride. The journal’s standing reflects the sustained efforts of its authors, reviewers, and editorial teams. Throughout its history, BIB has aimed to provide clarity in a rapidly evolving field: to synthesize, to compare, and to guide. This role remains as important as ever. As the volume of information continues to grow, the need for thoughtful, critical review becomes more—not less—pressing.

Bioinformatics has moved from a supporting role to a central position in the life sciences. Interdisciplinary collaboration is now routine, and training pathways have diversified accordingly. Yet familiar challenges remain: reproducibility, benchmarking, data quality, and the interpretation of increasingly complex models. These issues are not new, but their scale has grown with the field itself.

As BIB marks its 25th anniversary, the discipline stands at another point of transition. The integration of multi-omic data, the maturation of structure-based approaches, and the prospect of precision medicine all point towards a future in which computational insight is inseparable from biological understanding. Whether in the clinic or the laboratory, bioinformatics will continue to shape how we ask—and answer—biological questions.

Finally, I would like to acknowledge the community that has sustained the journal. BIB has always depended on individuals willing not only to generate new knowledge but also to explain it clearly and critically. That commitment to explanation—to making complex ideas accessible and usable—is, in my view, the journal’s enduring contribution.

